# Continuous Glucose Monitoring in Non-ICU Hospitalized Adults with Type 2 Diabetes: A Systematic Review

**DOI:** 10.3390/jcm15010034

**Published:** 2025-12-20

**Authors:** Darío Lara-Gálvez, Matilde Rubio-Almanza, Yolanda Aparicio-Ródenas, David Sanchis-Pascual, Pilar Masdeu-López-Cerón, Victor Pérez-Cervantes, Juan Francisco Merino-Torres

**Affiliations:** 1 Endocrinology and Nutrition Department, La Fe University and Polytechnic Hospital in Valencia, 46026 Valencia, Spain; rubio_matalm@gva.es (M.R.-A.); sanchis_davpas@gva.es (D.S.-P.); masdeu_pil@gva.es (P.M.-L.-C.); perez_viccer@gva.es (V.P.-C.); merino_jfr@gva.es (J.F.M.-T.); 2 Joint Research Unit on Endocrinology, Nutrition and Clinical Dietetics, Health Research Institute La Fe, 46026 Valencia, Spain; 3 Department of Pediatrics, Hospital Clínico Universitario de Valencia, 46010 Valencia, Spain; aparicio_yolrod@gva.es; 4 Department of Medicine, University of Valencia, 46010 Valencia, Spain

**Keywords:** continuous glucose monitoring, diabetes mellitus, type 2, hospitalization, inpatients, glycemic control, meta-analysis

## Abstract

**Background**: Continuous glucose monitoring (CGM) may overcome the limitations of intermittent point-of-care (POC) testing by providing real-time glucose trends and reducing treatment delays. This study aimed to evaluate the efficacy of CGM versus POC capillary testing in improving glycemic control among hospitalized non-Intensive Care Unit (non-ICU) adults with type 2 diabetes mellitus (T2DM). **Methods**: We conducted a systematic review and meta-analysis following PRISMA 2020 guidelines. We searched PubMed for randomized controlled trials published in English or Spanish that compared CGM with POC testing in hospitalized non-ICU adults ≥ 18 years old with T2DM and assessed risk of bias using the Cochrane RoB2 tool. The primary outcome was time in range (TIR). Secondary outcomes included time below range (TBR), time above range (TAR), mean glucose (MG), and glycemic variability (GV). **Results**: Seven randomized controlled trials (RCTs) including 1106 patients were analyzed. CGM significantly improved TIR (mean difference [MD] +8.15%; 95% confidence interval [CI]: +5.76, +10.55; *p* < 0.001) and reduced TAR > 180 mg/dL (MD −7.11%; 95% CI: −9.43, −4.78; *p* < 0.001) and TAR > 250 mg/dL (MD −3.96%; 95% CI: −5.29, −2.62; *p* < 0.001) compared with POC testing. MG also decreased with CGM (MD −11.27 mg/dL; 95% CI: −14.74, −7.81; *p* < 0.001). A modest reduction in TBR <70 mg/dL was observed (MD −0.29%; *p* < 0.001), whereas no significant differences were found for TBR < 54 mg/dL or GV. **Conclusions**: CGM improves inpatient glycemic control in non-ICU adults with type 2 diabetes, demonstrating advantages over POC testing across multiple randomized trials. However, further multicenter research is needed to clarify workflow implications, cost-effectiveness, and optimal implementation strategies.

## 1. Introduction

Patients with diabetes mellitus (DM) constitute a substantial proportion of individuals admitted to general hospital wards, accounting for more than one-third of non-ICU admissions [1,2]. Hyperglycemia is also frequently detected in patients without previously known diabetes, with reports indicating that roughly 12–25% develop elevated glucose levels during hospitalization [1,3,4]. Disturbances in glycemic control during an inpatient stay have been repeatedly associated with unfavorable outcomes—including a higher risk of infections and prolonged admissions—highlighting the need for reliable glucose monitoring strategies [4,5]. The Endocrine Society’s 2012 clinical practice guideline for non-critical care settings recommended maintaining blood glucose between 100 and 180 mg/dL in most hospitalized adults with either known diabetes or newly recognized hyperglycemia [6]. These recommendations were based on limited evidence drawn from heterogeneous studies, many of which were performed in specialized settings or extrapolated from critically ill populations [6,7,8]. Subsequent international guidelines, such as the American Diabetes Association (ADA) (updated annually, e.g., 2025 edition) have continued to endorse similar glycemic targets [9].

Management of inpatient diabetes has evolved considerably over the past decade. Therapeutic options have expanded, and new digital technologies have prompted renewed interest in strategies for monitoring glucose levels in hospital environments [10,11,12]. At that time, however, the available evidence base remained modest and heterogeneous regarding study designs, patient populations, and glucose-monitoring protocols. In ambulatory care, CGM has been widely adopted as standard practice for adults using basal insulin, multiple daily injections, or continuous subcutaneous insulin infusion, as well as for youth with type 1 diabetes, owing to its demonstrated benefits for glycemic control and treatment satisfaction [13,14,15]. In contrast, in most hospitals, capillary POC testing continues to be used as the routine monitoring method. While practical and familiar, POC testing is intermittent and may be affected by both analytical limitations and operator-dependent variability [9]. In parallel, CGM is emerging as a potential alternative modality in the hospital setting [16], but data supporting its use in inpatient settings remain limited.

Economic considerations have also influenced clinical adoption. CGM systems remain considerably more costly than standard POC testing, and formal cost-effectiveness evaluations for non-ICU inpatients are not yet available. Although modeling studies in outpatient populations suggest a favorable economic profile, analogous analyses for hospitalized adults are still lacking and have contributed to slower implementation [9,17,18].

During the COVID-19 pandemic, the U.S. Food and Drug Administration (FDA) issued temporary enforcement discretion permitting the inpatient use of certain factory-calibrated CGM systems, primarily to reduce staff exposure and preserve personal protective equipment [19]. This regulatory change led to a rapid expansion of inpatient CGM use and catalyzed research focused on accuracy, safety, workflow integration, and hybrid monitoring approaches combining CGM with POC testing. As a result, recent guidance from major professional societies—including the ADA and the American Association of Clinical Endocrinology (AACE)—now supports continued use of personal CGM devices during hospital stays when clinically appropriate, provided that critical decisions are confirmed with POC values [9,12].

Since then, several RCTs have been published evaluating inpatient CGM among non-ICU adults [20,21,22,23,24,25,26]. These studies differ considerably in their clinical and methodological features, including admission diagnoses, sensor types, calibration needs, alarm configurations, timing of sensor placement, monitoring duration, insulin-titration strategies, and concurrent treatments such as glucocorticoids or enteral nutrition.

Given this evolving landscape, the objective of this systematic review and meta-analysis is to synthesize evidence from randomized trials comparing CGM with capillary POC testing for inpatient glycemic management. Because inpatient TIR has been linked to clinically relevant outcomes—including infection rates, hemodynamic stability, and length of stay [15,27,28]—this review examines the extent to which CGM improves TIR, decreases hyperglycemia and hypoglycemia, and optimizes overall glycemic control in non-ICU adults with T2DM.

## 2. Methods

This systematic review and meta-analysis aimed to compare the performance of CGM with POC testing for managing inpatient dysglycemia in non-ICU adults with T2DM.

### 2.1. Primary and Secondary Outcomes

The primary outcome was defined as TIR (70–180 mg/dL). Secondary outcomes included TBR (<70 and <54 mg/dL), TAR (>180 and >250 mg/dL), MG, and GV, following international CGM interpretation consensus recommendations [29]. Additional information was collected on length of hospitalization, sensor accuracy based on the Mean Absolute Relative Difference (MARD), and the specific CGM system used.

### 2.2. Search Strategy

The review adhered to PRISMA 2020 recommendations for transparent reporting [30]. Although the review was not prospectively registered, we acknowledge the existence of an independently registered PROSPERO record with a related scope (CRD42024550000, 2024). This registration is unrelated to our group, and the present review was conducted independently.

PubMed was used as the primary database for this review, from 1 January 2000 to 31 January 2025, as it provides comprehensive coverage of major endocrinology, diabetology, and internal medicine journals and incorporates standardized MeSH terminology relevant to inpatient glycemic management. Although PubMed/MEDLINE was used as the primary electronic database, we also conducted complementary manual searches, including backward and forward citation screening of all included studies and relevant reviews, as well as checks of trial registries. These procedures did not identify additional eligible RCTs beyond those retrieved through PubMed. While it appears that existing inpatient CGM trials have been published in journals indexed in MEDLINE, reliance on a single primary database does not meet recommended multi-database standards and is therefore acknowledged as a methodological limitation.

The PubMed search was performed on 31 January 2025. The full search strategy combined free-text terms and MeSH headings as follows: (‘CGM’ OR ‘Continuous Glucose Monitoring’ OR ‘Glucose Monitoring, Continuous’ [MeSH]) AND (‘type 2 diabetes’ OR ‘Diabetes Mellitus, Type 2’ [MeSH]) AND (‘inpatient’ OR ‘Inpatients’ [MeSH] OR ‘Hospitalization’ [MeSH]).

The search was restricted to English and Spanish publications, which may introduce a potential language bias.

Study selection proceeded as follows: two reviewers independently screened titles and abstracts, followed by full-text assessment of potentially relevant articles. Any discrepancies were resolved through consultation with a third reviewer. Abstracts were included only when adequate outcome data were extractable. Conference abstracts were excluded due to insufficient methodological detail. Review articles were screened to identify additional eligible studies, and duplicates were removed.

### 2.3. Eligibility Criteria

#### 2.3.1. Inclusion Criteria

The following inclusion criteria were chosen to include studies in this study:

1. Population: studies in which the majority of participants had T2DM, or stress hyperglycemia (with or without previously known diabetes). This variability was recorded during data extraction and considered a potential source of clinical heterogeneity. Studies including a small proportion (<10%) of patients with type 1 diabetes (T1DM) were accepted if results were not stratified, and the population was predominantly T2DM.
2. Setting and intervention: studies conducted in non-ICU hospital environments in which CGM was used during at least part of the admission. Eligible populations included post-operative inpatients, individuals transferred from ICU to non-ICU wards before CGM initiation, patients receiving glucocorticoids, those scheduled for elective procedures, and those receiving artificial nutrition. Use of continuous subcutaneous insulin infusion (CSII) during admission was acceptable, provided this use was restricted to the inpatient setting and not part of long-term outpatient pump therapy.
3. Outcomes: studies that reported standard CGM-derived glycemic metrics relevant to inpatient monitoring (e.g., TIR, TAR, TBR, MG, or GV), in alignment with the objectives of this review.
4. Study design: only RCTs providing a direct comparison between CGM and capillary POC glucose testing.

#### 2.3.2. Exclusion Criteria

The exclusion criteria were as follows:

1. Studies involving exclusively adults with T1DM, pediatric patients, gestational diabetes, adults undergoing dialysis, adults admitted to ICU, and patients undergoing continuous intravenous insulin infusions.
2. Studies not involving hospitalized patients.
3. Studies that did not evaluate patients under insulin treatment, since inpatient glycemic protocols rely on scheduled insulin therapy and the effect of CGM cannot be meaningfully assessed in the absence of insulin-based management.
4. Studies using hybrid closed-loop systems.
5. Unpublished reports.
6. Animal or in vitro studies.

### 2.4. Data Collection and Quality Assessment

Two of the authors independently extracted data from all included studies using a standardized electronic form. A third author solved any disagreement between reviewers. Reviewers were not blinded to study authors or institutions.

We extracted data on patient characteristics (number of patients, mean age, female percentage, and details of intervention) and prespecified outcomes of interest, including MG, TAR > 180 mg/dL and 250 mg/dL, TBR < 70 mg/dL and <54 mg/dL, hospital length of stay, sensors’ accuracy assessed by MARD, type of CGM and 30-day readmission rate. When available, we also documented whether patients had established T2DM or stress-induced hyperglycemia, as this distinction was considered relevant for interpreting between-study differences. Only CGM-derived glycemic metrics (TIR, TBR, TAR, MG, and GV) were eligible for quantitative synthesis. All other extracted variables—such as hospital length of stay, 30-day readmission rates, and sensor accuracy—were summarized descriptively because they were not reported consistently or were not comparable across studies.

Risk of bias was evaluated using the Cochrane RoB 2 tool for randomized trials [31]. Two reviewers independently assessed each study, and discrepancies were resolved through discussion with a third reviewer. This tool evaluates five domains: bias arising from the randomization process, bias due to deviations from the intended interventions, bias due to missing outcome data, bias in measuring outcomes, and bias in selecting reported results. Visual summaries of the RoB2 assessment were included in Supplementary Table S1.

Certainty of evidence for each outcome was appraised using the Grading of Recommendations Assessment, Development and Evaluation (GRADE) approach [32]. Two reviewers independently evaluated the evidence across the five GRADE domains (risk of bias, inconsistency, indirectness, imprecision, and publication bias), starting from high certainty for randomized trials and downgrading as appropriate. The Summary of Findings table, including pooled estimates and 95% confidence intervals, is available in Supplementary Table S2.

### 2.5. Statistical Analysis

For studies included in the meta-analysis, mean values and standard deviations were extracted when reported. When only medians and interquartile ranges (IQRs) were provided, the mean was estimated as the midpoint of the IQR, serving as a practical approximation of the central tendency under small deviations from symmetry. The standard deviation (SD) was approximated using the conservative formula SD = (Q1 − mean)/−0.675, a method shown to provide reliable estimates across a variety of distributions, including moderately skewed data [33]. When a 95% confidence interval (CI) for the mean was reported, the SD was reconstructed from the CI using standard formulas.

Although all included trials defined TIR as 70–180 mg/dL, differences in sensor wear time, calibration requirements, and implementation protocols were documented during data extraction.

If a 95% confidence interval (CI) for the mean was reported, the SD was estimated using the formula: SD = ((UpperCI − LowerCI)/(2 × 1.96)) × √n. This expression follows directly from the standard form of a two-sided 95% CI for the sample mean: mean ± 1.96 × (SD/√n), and therefore the dependence on sample size is inherent to the definition of the standard error. This method is widely accepted for reconstructing SDs in meta-analyses when only CIs for means are available. For all continuous outcomes, the effect measure was the MD between the CGM and POC capillary testing groups.

The meta-analysis was conducted using the inverse-variance method with the restricted maximum-likelihood estimator (REML) for tau^2^, and confidence intervals for tau^2^ were calculated using the Q-profile method. Heterogeneity was assessed using the I^2^ statistic and tau^2^ which provides less biased estimates of between-study variance than classical methods, particularly when the number of studies is small to moderate.

Forest plots were constructed to summarize pooled MD (CGM minus capillary POC testing) for each continuous outcome. For each trial, we calculated the difference in means between the CGM and control groups and its standard error using the reported or reconstructed means and standard deviations. These effect sizes were then combined using inverse-variance random-effects models. When a given endpoint was reported only for CGM users or with non-comparable definitions across treatment arms, results were summarized descriptively without entering a comparative meta-analysis.

Publication bias was explored using funnel plots and the Begg–Mazumdar rank correlation test. Given the small number of studies per outcome, the assessment of publication bias is methodologically appropriate but inherently limited, and any observed asymmetry should be interpreted with caution. Egger’s test and trim-and-fill procedures were not performed due to their unreliability with fewer than ten studies [34].

Subgroup analyses and meta-regression were not performed because the number of available randomized trials was insufficient to support these methods. According to Cochrane and PRISMA methodological guidance, subgroup or regression analyses require at least 10 studies to produce stable estimates and avoid spurious associations [30,35]. In this review, only seven studies were eligible overall, and most potential subgroups (e.g., sensor type, insulin regimen, alarm thresholds) contained fewer than three trials, making such analyses statistically unreliable and methodologically inappropriate.

## 3. Results

### 3.1. Study Selection

**Figure 1:**
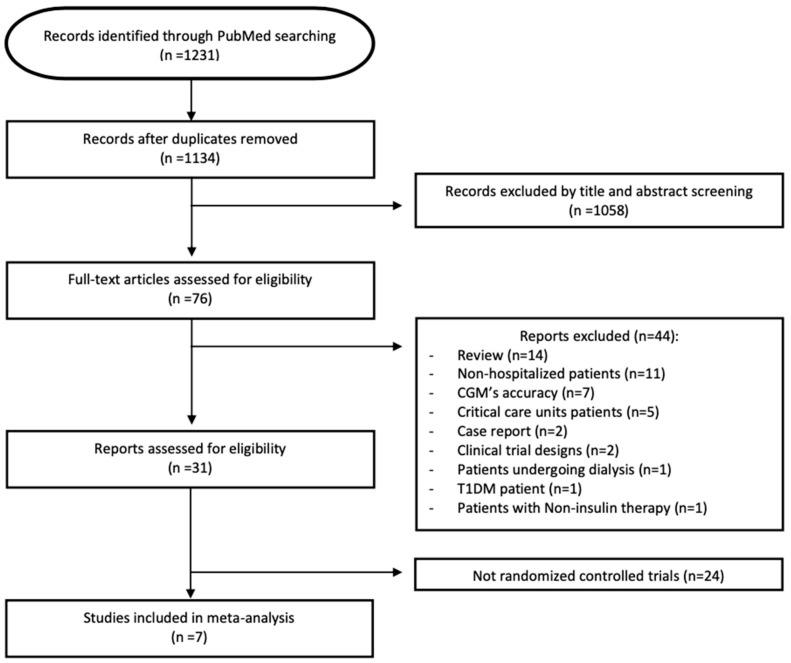
2020 flow diagram for study selection. The database search was performed on 31 January 2025 using PubMed as the data source.

The electronic search initially yielded 1328 records (Figure 1). After removal of 95 duplicates, 1233 unique citations remained for screening. Of these, 1158 were excluded based on title and abstract review, and 75 full-text articles were assessed for eligibility.

Among the 75 full texts, 44 were excluded for the following reasons: review articles (n = 14), studies not conducted in hospitalized populations (n = 11), investigations limited to CGM accuracy (n = 7), trials performed exclusively in ICU settings (n = 5), case reports (n = 2), reports describing RCT designs without outcome data (n = 2), a study conducted in patients on dialysis (n = 1), a trial restricted to individuals with T1DM (n = 1), and one study examining oral antidiabetic therapies rather than CGM (n = 1). The remaining 31 articles compared CGM and capillary glucose monitoring either directly or indirectly, though with substantial heterogeneity. However, to ensure methodological consistency, only RCTs were ultimately selected, resulting in a final sample of seven RCTs.

### 3.2. Risk of Bias

Risk-of-bias assessments for the included RCTs are provided in the Supplementary Material. Using the Cochrane RoB 2 tool (RoB 2) [31], two studies (Singh et al. [20] and Idrees et al. [21]) were judged to have low risk of bias and demonstrated robust trial conduct. Four studies (Fortmann et al. [22]; Spanakis et al. [23], Wang et al. [24] and Olsen et al. [25]) were rated as having some concerns, largely related to open-label designs, deviations from intended interventions, or selective reporting. Thabit et al. [26] had a high risk of bias due to deviations in intervention adherence and lack of blinding. Overall, these findings indicate that important methodological limitations were present in several trials, and pooled estimates should therefore be interpreted with appropriate caution.

### 3.3. Baseline Characteristics of Included Studies

The baseline characteristics of the studies included in the meta-analysis are summarized in Table 1.

Five trials (Fortmann, Singh, Spanakis, Wang, and Olsen) enrolled patients treated with a basal–bolus insulin regimen throughout the study period. In the trial by Idrees et al. [21] (n = 97), approximately one-quarter of participants continued oral antidiabetic drugs, with comparable use between study arms. Thabit et al. [26] (n = 24) included individuals receiving both insulin and oral agents.

A total of 1106 patients were analyzed, of whom 656 were men (59.3%). The weighted mean age across the studies was 69.9 years (SD: 15.6). One study reported hospital 30-day readmission rates (Olsen et al. [25]), with no differences between groups. None of the included trials collected or reported patient satisfaction data. Safety outcomes and in-hospital complications were reported infrequently and in a heterogeneous manner across trials. Fortmann et al. [22] and Wang et al [24]. described no qualifying or serious adverse events, whereas Spanakis et al. [23] reported only a small number of minor bleeding episodes and sensor malfunctions, with no statistically significant differences between groups. The remaining trials did not systematically report CGM-specific complications. None of the randomized studies evaluated patient satisfaction or other patient-reported outcomes, and HbA1c values, when available, were reported only as baseline descriptors rather than predefined inpatient efficacy endpoints.

Different CGM systems were employed, including Dexcom G6, Dexcom G7, and Guardian 3 sensors. Reported MARD values were 9.8% for Dexcom G6 [36,37], 8.2–9.1% for Dexcom G7, depending on sensor placement (arm versus abdomen) [38], and 8.7–9.6% for Guardian 3, with performance varying according to calibration frequency [39]. The trials were conducted in the United States, Australia, China, Denmark, and the United Kingdom, providing a geographically diverse sample.

### 3.4. Synthesis of Results

**Figure 2:**
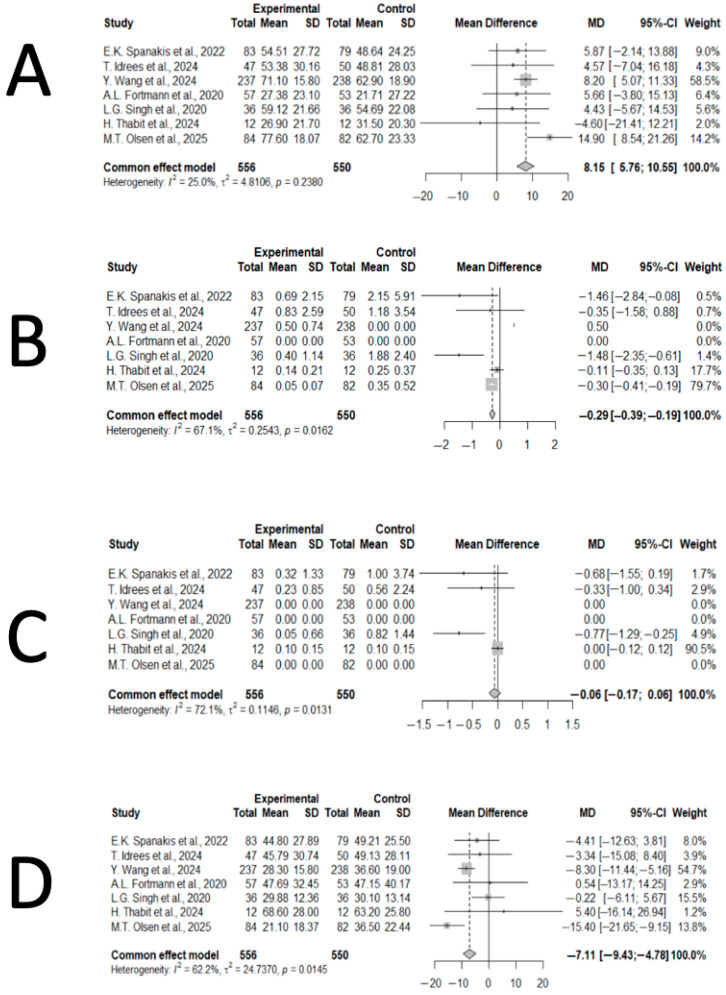
Forest plots showing the pooled effects of CGM on TIR, TBR, and TAR outcomes [20,21,22,23,24,25,26]. (**A**). Forest plot illustrating the effect of CGM on the percentage of TIR (70–180 mg/dL) during hospitalization. Most studies favored CGM over POC testing, with a consistent improvement in glycemic control. (**B**). Forest plot showing the effect of CGM use on TBR < 70 mg/dL. Pooled analysis demonstrated a reduction in hypoglycemia, though heterogeneity across studies was substantial (I^2^ = 67.1%). (**C**). Forest plot showing the effect of CGM use TBR < 54 mg/dL. No significant difference was observed between CGM and POC testing; however, variability across studies was considerable (I^2^ = 72.1%). (**D**). Forest plot showing the effect of CGM use on TAR > 180 mg/dL. CGM significantly reduced hyperglycemia (MD = −7.11, 95% CI: −9.43 to −4.78, *p* < 0.001), with moderate heterogeneity (I^2^ = 62.2%).

Quantitative analyses focused on CGM-derived glycemic metrics, which were the most consistently reported outcomes across trials. The meta-analysis of seven RCTs comprising 1106 participants (556 assigned to CGM and 550 to POC testing) demonstrated a significant increase in TIR (70–180 mg/dL) with CGM use. The pooled MD was +8.15 percentage points in favor of CGM (95% CI: 5.76–10.55; *p* < 0.001). Between-study heterogeneity was low (I^2^ = 25%, *p* = 0.24), supporting the consistency of this effect (Figure 2).

For TBR < 70 mg/dL, CGM was associated with a small but statistically significant reduction in hypoglycemia (MD = −0.29 percentage points, 95% CI: −0.39 to −0.19; *p* < 0.001). Heterogeneity was moderate to high (I^2^ = 67.1%, *p* = 0.016), suggesting that differences in patient profiles or insulin-management strategies may have influenced this outcome (Figure 2). In contrast, for TBR < 54 mg/dL, no significant difference between groups was observed (MD = −0.06 percentage points, 95% CI: −0.17 to 0.06; *p* = 0.3128), and heterogeneity was high (I^2^ = 72.1%, *p* = 0.013), reflecting variability in populations, insulin protocols, and monitoring durations (Figure 2).

**Figure 3:**
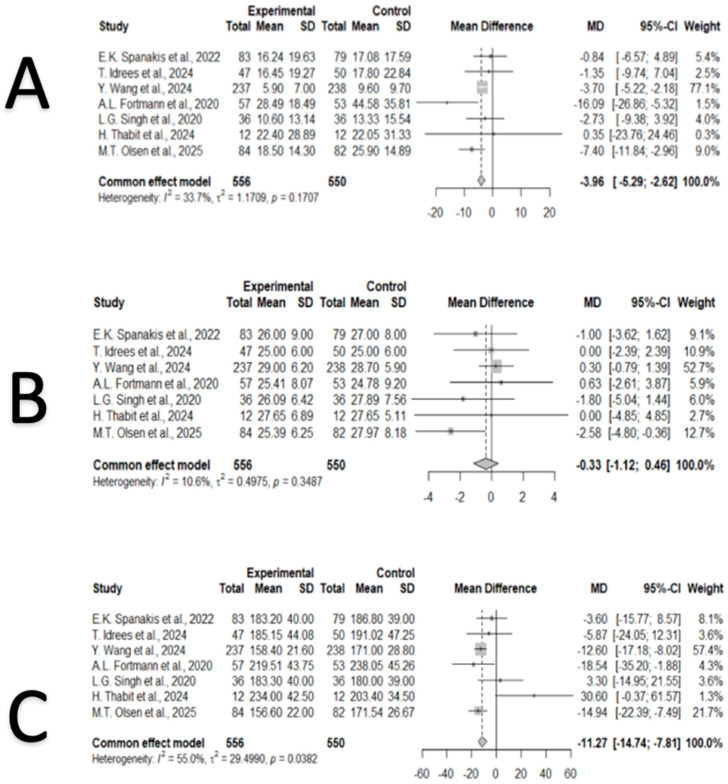
Forest plots showing the pooled effects of CGM on hyperglycemia, GV, and MG levels [20,21,22,23,24,25,26]. (**A**). Forest plot showing the effect of CGM use on TAR > 250 mg/dL). CGM was associated with significantly lower exposure to glucose levels above 250 mg/dL (MD = −3.96, 95% CI: −5.29 to −2.62, *p* < 0.001). Results were homogeneous across studies (I^2^ = 33.7%). (**B**). Forest plot showing the effect of CGM use on GV, measured by CV. No significant change in GV was observed (MD = −0.33, 95% CI: −1.12 to 0.46, *p* = 0.41), with low heterogeneity (I^2^ = 10.6%). (**C**). Forest plot showing the effect of CGM use on MG levels. CGM significantly reduced MG (MD = −11.27 mg/dL, 95% CI: −14.74 to −7.81, *p* < 0.001), with moderate heterogeneity (I^2^ = 55.0%).

Regarding hyperglycemia, CGM use was associated with lower TAR > 180 mg/dL compared with POC testing (MD = −7.11 percentage points, 95% CI: −9.43 to −4.78; *p* < 0.001). Moderate heterogeneity was present (I^2^ = 62.2%, *p* = 0.015) (Figure 2). For TAR > 250 mg/dL, CGM similarly reduced time spent above this threshold (MD = −3.96 percentage points, 95% CI: −5.29 to −2.62; *p* < 0.001), with low-to-moderate heterogeneity (I^2^ = 33.7%, *p* = 0.17) (Figure 3).

No significant difference was observed in GV by CGM use, assessed by the coefficient of variation (CV) (MD = −0.33 percentage points, 95% CI: −1.12 to 0.46, *p* = 0.413). Heterogeneity was minimal (I^2^ = 0%, *p* = 0.35), supporting the consistency of this finding across studies. (Figure 3).

MG levels were significantly lower with CGM use (MD = −11.27 mg/dL, 95% CI: −14.74 to −7.81, *p* < 0.001). Moderate heterogeneity was observed (I^2^ = 55.0%, *p* = 0.038), indicating some variability across studies (Figure 3). In summary, the included RCTs showed consistent numerical improvements in several glycemic metrics—such as TIR, hyperglycemia thresholds, and MG—in the CGM groups compared with POC testing, whereas effects on hypoglycemia and GV were small or not statistically significant.

### 3.5. Publication Bias

**Figure 4:**
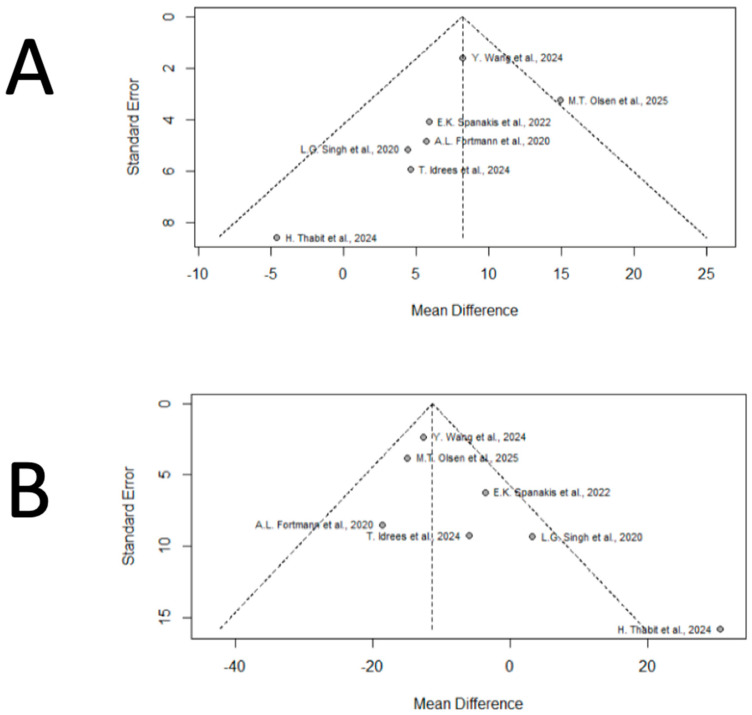
Funnel plots assessing publication bias for primary outcomes [20,21,22,23,24,25,26]. (**A**). Funnel plot assessing publication bias for the outcome TIR. The plot shows visual asymmetry, with a relative absence of small studies showing null or negative effects, suggesting potential publication bias. This observation was supported by Begg and Mazumdar’s rank correlation test (*p* = 0.0243). (**B**). Funnel plot assessing publication bias for the outcome MG. Slight asymmetry was observed, indicating a marginal suggestion of publication bias (*p* = 0.051), possibly reflecting that smaller studies may have overestimated the true effect size.

Funnel plot analysis and Begg and Mazumdar’s rank correlation test suggested the presence of publication bias for TIR (*p* = 0.0243) (Figure 4) and for a marginal indication of bias for MG (*p* = 0.051) (Figure 4), suggesting that smaller studies may have overestimated the true effect size. No evidence of publication bias was detected for other variables. However, given that each meta-analysis included fewer than ten studies, these assessments must be interpreted with caution. With such small numbers, both funnel plots and rank-based tests have very low statistical power and are prone to false-positive or inconclusive findings. For this reason, regression-based approaches such as Egger’s test were not applied, as they are considered statistically unreliable under these conditions. Similarly, trim-and-fill procedures were not performed because they are highly unstable and tend to overcorrect when evidence is sparse [30,34]. Therefore, while small-study effects cannot be fully excluded, the available data are insufficient to support a definitive evaluation of publication bias.

### 3.6. Consistency

Consistency of findings varied across outcomes. For TIR, TAR (>180 mg/dL and >250 mg/dL), and MG, heterogeneity was low to moderate (I^2^ < 40% to 60%), and all analyses consistently favored the use of CGM, indicating a stable and directionally uniform effect across studies. These outcomes were also supported by narrow confidence intervals, reinforcing the robustness of the findings.

In contrast, TBR < 70 mg/dL, TBR < 54 mg/dL, and GV showed higher heterogeneity (I^2^ > 60%), reflecting differences in study design, patient populations, insulin regimens, CGM alarm thresholds, and ward management protocols. Several studies reported extremely low or zero values for hypoglycemia-related outcomes, which further contributed to variability and reduced precision.

Given the small number of eligible studies and the limited number of trials contributing to each potential subgroup, formal subgroup analyses or meta-regression could not be meaningfully undertaken.

## 4. Discussion

This work provides a consolidated evaluation of how CGM compares with routine capillary glucose testing for glycemic management in non-ICU hospitalized adults with T2DM. The seven RCTs [20,21,22,23,24,25,26]) included—spanning five countries and a variety of ward settings—offer a broad representation of current inpatient practice. In total, 1106 individuals contributed data to the quantitative analyses, making this synthesis one of the most extensive evaluations of inpatient CGM performance using standardized glycemic metrics.

One study (Wang et al.) was carried out in a long-term or chronic-care unit and enrolled only patients managed with short-term CSII [24]. Although this setting differs from conventional non-ICU wards, the trial evaluated the same comparison (CGM vs. POC testing) and reported the same glycemic endpoints as the other RCTs. Its structured nurse-directed insulin titration protocol closely resembles practices in many inpatient units. For these reasons, the study was retained but classified as contributing indirectness in the GRADE assessment, and its specific context should be considered when generalizing the overall findings.

Across trials, participants were older adults (average age close to 70 years) and predominantly received inpatient insulin therapy. The clinical contexts varied—from perioperative care to steroid-induced hyperglycemia—reflecting the heterogeneity typical of medical and surgical wards. Most studies used basal-bolus regimens, while one focused exclusively on short-term CSII and two permitted limited use of oral antidiabetic drugs. These characteristics shape GV during hospitalization and likely contribute to differences in effect sizes across studies. However, the small number of eligible RCTs precluded any meaningful exploration of effect modifiers through subgroup analysis or meta-regression.

### 4.1. Principal Findings

The pooled estimates indicate that CGM use in non-ICU hospitalized adults leads to noticeable improvements in several glycemic indicators. The most consistent signal was the increase in TIR, with a mean improvement of 8.15 percentage points. Considering that each 1% rise in TIR corresponds to approximately 14.4 additional minutes per day in the target range [26] the overall change observed here is equivalent to nearly two hours of extra time spent within recommended glucose levels. This level of improvement exceeds the 5% threshold widely regarded as clinically relevant in diabetes care [27,28].

CGM also reduced exposure to hyperglycemia at both TAR > 180 mg/dL and TAR > 250 mg/dL thresholds, reinforcing the potential benefit of continuous data for anticipating and correcting glucose trends upward.

Reductions in hypoglycemia were more modest and were statistically significant only for TBR < 70 mg/dL. The absence of a measurable effect for TBR < 54 mg/dL likely reflects the extremely low event rates reported in several trials. Improvements in MG further support the capacity of CGM to reduce overall glycemic burden, whereas GV—assessed as CV—remained largely unchanged. The variability in TBR and GV outcomes, together with moderate heterogeneity, suggests that site-level differences in clinical practice and insulin-titration responsiveness may modulate the impact of CGM on these endpoints.

### 4.2. Comparison with Previous Literature

The results of the included studies are aligned with previous evidence from outpatient settings, where CGM is widely accepted as the standard of care for insulin-treated diabetes [31], and increasingly recommended in perioperative and long-term care environments [32,33]. However, in the inpatient setting, the literature remains sparse. Prior studies—including those analyzed here—suggest that CGM may offer improved detection of glycemic excursions and may prevent unnoticed nocturnal hypoglycemia. However, not all trials demonstrated consistent clinical superiority. For example, Spanakis et al. [23] and Thabit et al. [26] found no significant differences in TIR, possibly due to limited responsiveness of non-specialized teams to CGM data or the absence of structured insulin titration protocols. Olsen et al. [25] and Wang et al. [24] reported the most favorable outcomes, likely reflecting structured insulin titration protocols and active involvement of diabetes-specialized teams. Singh et al. [20] emphasized the utility of CGM in identifying and reducing hypoglycemic episodes, while Fortmann et al. [22] showed that CGM was safe, feasible, and associated with higher dysglycemia detection. Idrees et al. [21], using blinded CGM, demonstrated that many glycemic events remained undetected by standard POC, underscoring the diagnostic advantage of CGM even when not actively used for decision-making.

Despite the growing interest in inpatient CGM, randomized controlled trials in non-ICU hospital settings remain scarce, underscoring the need for more conclusive data in this population. Our meta-analysis builds upon prior research by offering a more comprehensive and statistically robust evaluation, revealing significant improvements in key glycemic endpoints. Overall, CGM was generally associated with higher TIR and lower TAR, while benefits in TBR were modest and more variable across studies. Those are metrics increasingly recognized by international guidelines as clinically meaningful indicators of glycemic control [15].

### 4.3. Mechanistic Interpretation

The observed improvements may be related to the continuous, real-time glucose data provided by CGM systems, which enable more precise and timely insulin adjustments compared with intermittent POC testing. Moreover, CGM systems allow early identification of glycemic trends, supporting preemptive interventions that may help reduce the frequency or duration of glycemic excursions and mitigate the duration and severity of hypoglycemic episodes [40].

The absence of significant differences in GV, measured by CV and SD, may reflect the underlying heterogeneity across studies, including differences in diabetes duration, baseline therapies, and reasons for hospitalization. Likewise, the limited and inconsistent reporting of hypoglycemia—together with short monitoring periods, conservative insulin titration strategies, and sparse or zero-event data in several trials—likely contributed to the modest or non-significant effects on TBR < 70 mg/dL and <54 mg/dL. This variability contributes to the moderate-to-low certainty ratings for hypoglycemia-related outcomes.

Differences in CGM alarm thresholds, alert workflows, and the integration of alerts into clinical practice likely contributed to the heterogeneity observed in TBR < 70 mg/dL and TAR > 180 mg/dL. As summarized in Supplementary Table S3, some trials employed lower hypoglycemia alarm settings or more proactive alert management, whereas others used higher thresholds or relied primarily on confirmatory POC testing. Additional site-level factors—such as staffing ratios, diabetes team involvement, and differences in insulin titration protocols and baseline hypoglycemia risk—further reduced comparability across studies. Collectively, these elements influence the frequency of detected events and plausibly explain the moderate-to-high heterogeneity observed in hypoglycemia-related outcomes.

CGM implementation has also been associated with reductions in nursing workload for glucose monitoring and insulin titration—an aspect highlighted in several of the included trials. Such efficiencies gained particular relevance during the COVID-19 pandemic, when CGM was used to reduce staff exposure and preserve protective equipment [20]. Future studies evaluating nursing resource utilization with CGM versus POC capillary glucose testing could provide critical insights into the cost-effectiveness of inpatient CGM and may catalyze broader adoption in hospital settings.

Taken together, these findings suggest that the observed benefits likely arise from the integration of CGM data into structured inpatient workflows rather than from CGM use in isolation. Trials demonstrating the largest improvements (such as Olsen et al. [25] and Wang et al. [24]) were those incorporating protocol-driven insulin titration, predefined alarm-response algorithms, and active involvement of diabetes specialists, whereas studies with limited clinical responsiveness to CGM information (e.g., Spanakis et al. [23], Thabit et al. [26]) showed attenuated or null effects. This distinction is essential from an implementation standpoint: continuous glucose data can only translate into improved glycemic outcomes when embedded within organizational processes capable of acting on these data in real time.

### 4.4. Future Directions

The available evidence suggests a potentially expanding role of CGM during hospitalization in improving glycemic control and safety in non-ICU patients with diabetes. However, its potential extends beyond the inpatient setting. Transition of care after hospital discharge remains a high-risk period, particularly for insulin-treated individuals with T2D, who often experience therapeutic inertia and remain vulnerable to hypoglycemia, as previously reported by Tian et al. [41], a systematic review in which only one relevant article assessing CGM use at discharge was found, highlighting the limited evidence base and multiple barriers to implementation in this population. Despite extensive evidence in T1DM supporting the early use of technology and structured CGM-based care from the outset, comparable data in T2D remain scarce. Notably, a pilot RCT by Umpierrez et al. [42] was conducted to compare the safety, efficacy, and healthcare utilization between the use of CGM and POC testing in insulin-treated patients with T2D after hospital discharge. Although no statistically significant differences were observed, the study showed a clear trend toward improved glycemic control and more efficient insulin use in the CGM group compared to standard POC monitoring. The evidence from this meta-analysis highlights the need to further examine how CGM could facilitate safer and more effective transitions of care in T2D, a population with distinct clinical profiles and therapeutic needs. Future research should prioritize larger, multicenter trials evaluating CGM-based strategies in this setting, with a focus on glycemic and patient-centered outcomes, healthcare utilization, and long-term safety. Such efforts are essential to bridge the current knowledge gap and translate inpatient technological advances into sustained outpatient benefits.

### 4.5. Sources of Bias and Limitations

Several issues should be considered when interpreting the findings of this meta-analysis. To begin with, the review synthesizes evidence from a small number of randomized trials, reflecting the early development of research on inpatient CGM. As in any meta-analysis, the results inevitably inherit methodological constraints and biases present in the individual studies. Variability in key clinical features—such as duration of diabetes, reasons for admission, background glucose-lowering therapies, and ward-level glycemic management approaches—likely contributed to differences in effect sizes across trials.

A further source of variation was the substantial clinical and methodological heterogeneity observed among the included studies. Differences in inpatient glycemic protocols, insulin-titration practices, and baseline metabolic status created diverse clinical contexts that complicate direct comparisons. In addition, CGM-related characteristics varied considerably across trials: multiple sensor platforms were used (Dexcom G6, Dexcom G7, Guardian Sensor 3), and studies differed in calibration requirements, alarm thresholds, alert-handling procedures, and monitoring frequency. These device- and workflow-related elements influence the detection of glycemic excursions and therefore may have shaped the magnitude of the pooled effects. Among these factors, variation in device accuracy, differences in alarm-setting strategies, and the degree of clinician responsiveness to alerts appear most likely to have influenced the pooled treatment effects.

Although some outcomes—particularly TBR < 70 mg/dL and TAR > 180 mg/dL—showed moderate-to-high heterogeneity, the limited number of eligible RCTs precluded meaningful subgroup or meta-regression analyses. Methodological guidance from both Cochrane and PRISMA emphasizes that such analyses typically require at least ten studies per comparison to avoid unstable or misleading results. Given the insufficient number of studies and the sparse representation of potential subgroups (e.g., sensor brand, insulin regimen, alarm configuration), heterogeneity was interpreted descriptively. Contextual explanations were provided narratively rather than through formal stratification.

Another limitation relates to differences in inpatient treatment regimens. Five studies enrolled exclusively insulin-treated patients, whereas two trials included individuals receiving mixed insulin and non-insulin therapies. These differences could influence glycemic patterns and responsiveness to CGM-guided management, contributing to variability across studies.

Several clinically relevant endpoints—such as length of hospital stay, 30-day readmission rates, and device failures—were seldom reported and, when reported, lacked consistency across trials. This restricts the ability to evaluate important patient-centered or operational outcomes. Additionally, the small number of available studies limits the interpretability of publication bias assessments; although Begg’s test and visual inspection of funnel plots were performed, regression-based methods such as Egger’s test are not recommended when fewer than ten studies are available. Consequently, any potential small-study effects or selective non-publication cannot be formally evaluated with confidence. Moreover, several studies reported only medians or confidence intervals, requiring reconstruction of means and standard deviations. While this approach is methodologically accepted, it may introduce additional uncertainty and affect the precision of the pooled estimates. This limitation should be taken into account when interpreting the results.

Language and database restrictions may also have introduced bias. In addition, the search relied primarily on PubMed, which may reduce the completeness of study retrieval. Although complementary manual searches were performed—including citation screening and examination of trial registries—these approaches cannot fully substitute for multi-database searches. While available evidence suggests that most inpatient CGM randomized trials are published in journals indexed in MEDLINE, it cannot be assumed with certainty that no eligible studies exist outside this database. Therefore, the reliance on a single primary database should be considered a methodological limitation.

One trial—Wang et al.’s [24]—contributed a disproportionately large sample size. Also, it was conducted in a single tertiary endocrinology ward, exclusively enrolled patients managed with short-term CSII, and used the Guardian™ Sensor 3. This highly structured clinical environment, combined with a more homogeneous patient mix and intensive insulin titration workflow, may limit the generalizability of the pooled estimates.

Finally, although individuals with T1DM were generally excluded, two trials included small proportions of participants with T1DM. These individuals represented a minority of the sample, and results were not stratified by diabetes type, but their inclusion introduces an element of indirectness that should be considered when interpreting the findings.

## 5. Conclusions

In conclusion, this meta-analysis indicates that adding real-time CGM to standard POC glucose testing is associated with improved inpatient glycemic control in non-ICU adults with T2DM. Nonetheless, due to the limited number of studies [7], heterogeneity in clinical protocols, and underreporting of patient-centered outcomes, further research is warranted to confirm these findings and support broader implementation.

## Figures and Tables

**Table 1:** Baseline characteristics of studies included in the meta-analysis.

Study	Group	Paients per Group	Overall Number of Patients	% Men	Mean Age (Years) (SD)	% T2DM	Sensor	% TIR (SD)	% TBR < 70 (SD)	% TBR < 55 (SD)	% TAR > 180 (SD)	% TAR > 250 (SD)	GV (CV) (SD)	MG (mg/dL) (SD)	Mean Hospitalized Time (Days)	30-Day Readmissions IRR (95% CI)
Fortmann et al. (2020) [22]	Active	57	110	47.4	62.9 (14.0)	100	DEXCOM G6	27.4 (23.1)	0.0 (0.0)	0.0 (0.0)	47.7 (32.5)	28.5 (18.5)	25.4 (8.1)	219.5 (43.8)	6 (4.4)	NA
	Control	53		43.4	60.9 (12.4)	100		21.7 (27.2)	0.0 (0.0)	0.0 (0.0)	47.2 (40.2)	44.6 (35.8)	24.8 (9.2)	238.1 (45.3)	5.5 (3.7)	
Singh et al. (2020) [20]	Active	36	72	91.7	68 (9.0)	100	DEXCOM G6	59.1 (21.7)	0.4 (1.1)	0.1 (0.7)	29.9 (12.4)	10.6 (13.4)	26.1 (6.4)	183.3 (40.0)	NA	NA
	Control	36		94.4	68 (10.0)	100		54.7 (22.1))	1.9 (2.4)	0.8 (1.4)	30.1 (13.1)	13.3 (15.5)	27.9 (7.6)	180.0 (39.0)		
Spanakis et al. (2022) [23]	Active	83	162	58	57.3 (12.3)	88	DEXCOM G6	54.5 (27.7)	0.7 (2.1)	0.3 (1.3)	16.2 (19.6)	16.2 (19.6)	26.0 (9.0)	183.2 (40.0)	NA	NA
	Control	79		63	55.1 (9.3)	91		48.6 (24.3)	2.2 (5.9)	1.0 (3.7)	17.1 (17.6)	17.1 (17.6)	27.0 (8.0)	186.8 (39.0)		
Idrees et al. (2024) [21]	Active	47	97	38	74.9 (11.7)	100	DEXCOM G6	53.4 (30.2)	0.8 (2.6)	0.2 (0.9)	16.5 (19.3)	16.2 (19.6)	25.0 (6.0)	185.2 (44.1)	1.0 (12.4)	NA
	Control	50		28	74.5 (10.6)	100		48.8 (28.0)	1.2 (3.5)	0.6 (2.2)	17.8 (22.8)	17.1 (17.6)	25.0 (6.0)	191.0 (47.3)	1.5 (3)	
Wang et al. (2024) [24]	Active	237	475	57	71.1 (15.8)	89	GUARDIAN SENSOR 3	71.1 (15.8)	0.0 (0.0)	0.0 (0.0)	5.9 (7.0)	5.9 (7.0)	29.0 (6.2)	158.4 (21.6)	6.7 (0.8)	
	Control	238		63.4	62.9 (18.9)	92		62.9 (18.9)	0.0 (0.0)	0.0 (0.0)	9.6 (9.7)	9.6 (9.7)	28.7 (5.9)	171.0 (28.8)	6.7 (0.8)	NA
Thabit et al. (2024) [26]	Active	12	24	76	62.1 (9.0)	100	DEXCOM G7	26.9 (21.7)	0.1 (0.2)	0.1 (0.2)	22.4 (8.9)	22.4 (28.9)	27.7 (6.9)	234.0 (42.5)	6.5 (5.2)	NA
	Control	12				100		31.5 (20.3)	0.3 (0.4)	0.1 (0.2	22.1 (31.3)	22.1 (31.3)	27.7 (5.1)	203.4 (34.5)	7 (4.4)	
Olsen et al. (2025) [25]	Active	84	166	61.9	76.6 (9.5)	100	DEXCOM G6	77.6 (24.4)	0.0 (0.1)	0.0 (0.0)	21.1 (24.8)	1.3 (5.6)	25.39 (6.25)	8.7 (1.7)	5.0 (5.0)	0.88 (0.74–1.04)
	Control	82		65.9	75.5 (10.1)	100		62.7 (31.5)	0.0 (0.7)	0.0 (0.0)	36.5 (30.3)	11.2 (16.1)	27.97 (8.18)	9.5 (2.0)	5.0 (4.3)	1.00 (Reference)

Legend: N, number; %, percentage; SD, standard deviation; TIR, Time in range; TBR, Time below range; TAR, Time above range; GV, glycemic variability; CV, Coefficient of Variation; T2DM, Type 2 diabetes; NA, not available.

## Data Availability

The datasets generated and analyzed during the present study are available from the corresponding author upon reasonable request.

## Supplementary Materials

The following supporting information can be downloaded at: https://www.mdpi.com/article/10.3390/jcm15010034/s1, Table S1: Summary table of version 2 of the Cochrane Risk of Bias tool (RoB 2) for each analyzed article. Table S2: GRADE Summary of Findings for the Effect of Continuous Glucose Monitoring (CGM) versus Point-of-Care (POC) Testing in Hospitalized Adults with Type 2 Diabetes. Table S3: Glycemic thresholds for CGM-triggered alarms in the intervention arms of included studies. Thresholds for hypoglycemia and hyperglycemia alarms varied substantially across studies included in the meta-analysis. Values are shown in both mg/dL and mmol/L. File S1: The PRISMA 2020 checklist.
