# Supplementary material for: Continuous Glucose Monitoring in Non-ICU Hospitalized Adults with Type 2 Diabetes: A Systematic Review
Source: J Clin Med. 2025 Dec 20;15(1):34. doi: 10.3390/jcm15010034 (PMC12786850; doi:10.3390/jcm15010034)
Supplement: Supplementary file 1 [file jcm-15-00034-s001.zip › jcm-4018749-supplementary Tables.pdf]

Supplementary Table S1. Summary table of version 2 of the Cochrane Risk of Bias tool (RoB 2) for each analyzed article.

	Randomization Process	Deviations from intended intervention	Missing outcome data	Measurement of outcome	Selection of reported result	Overall risk of bias
Fortmann et al. (2020)	Low risk	Some concerns	Low risk	Low risk	Some concerns	Some concerns
Singh et al. (2020)	Low risk	Low risk	Low risk	Some concerns	Low risk	Low risk
Spanakis et al (2022)	Some concerns	Low risk	Some concerns	Some concerns	Low risk	Some concerns
Idrees et al. (2024)	Low risk	Low risk	Low risk	Low risk	Low risk	Low risk
Wang et al. (2024)	Some concerns	Some concerns	Low risk	Some concerns	Some concerns	Some concerns
Thabit et al. (2024)	Some concerns	Some concerns	Some concerns	High risk	Some concerns	High risk
Olsen et al (2025)	Low risk	Some concerns	Low risk	Low risk	Some concerns	Some concerns

Supplementary Table S2. GRADE Summary of Findings for the Effect of Continuous Glucose Monitoring (CGM) versus Point-of-Care (POC) Testing in Hospitalized Adults with Type 2 Diabetes.

**Outcome**	**Participants (studies)***	**Study design**	**Risk of bias**	**Inconsistency**	**Indirectness**	**Imprecision**	**Other considerations**	**Effect (MD, 95% CI)****	**Certainty of the evidence (GRADE)**
**Time in Range (70–180 mg/dL)**	1106 (7 RCTs)	RCTs	Not serious	Not serious (I^2 = 25%)	Not serious	Not serious	None	**MD +8.15 percentage points** (95% CI 5.76 to 10.55)	**High**
**Time Below Range <70 mg/dL**	≈930 (5 RCTs)	RCTs	Not serious	**Serious** (I^2 = 67%)	Not serious	Not serious	None	**MD –0.29 percentage points** (95% CI –0.39 to –0.19)	**Moderate**
**Severe hypoglycaemia <54 mg/dL**	≈650 (4 RCTs)	RCTs	Not serious	**Serious** (I^2 ≈ 72%)	**Serious** (non-uniform definitions, sparse data)	**Serious** (CI includes no effect; very few events)	None	**MD –0.06 percentage points** (95% CI –0.17 to 0.06)	**Low**
**Time Above Range >180 mg/dL**	1106 (7 RCTs)	RCTs	Not serious	**Serious** (I^2 = 62%)	Not serious	Not serious	None	**MD –7.11 percentage points** (95% CI –9.43 to –4.78)	**Moderate**
**Time Above Range >250 mg/dL**	1106 (7 RCTs)	RCTs	Not serious	Not serious (I^2 = 34%)	Not serious	Not serious	None	**MD –3.96 percentage points** (95% CI –5.29 to –2.62)	**High**
**Mean glucose (mg/dL)**	1106 (7 RCTs)	RCTs	Not serious	Not serious (I^2 = 55%; same direction of effect)	Not serious	Not serious	None	**MD –11.27 mg/dL** (95% CI –14.74 to –7.81)	**High**
**Glycaemic variability (CV, %)**	1106 (7 RCTs; 4–5 with usable data)	RCTs	Not serious	Not serious (I^2 = 10.6%)	**Serious** (different definitions / calculation methods; incomplete reporting)	**Serious** (CI includes no important effect)	None	**MD –0.33 percentage points** (95% CI –1.12 to 0.46)	**Low**

Legend. CGM: continuous glucose monitoring; POC: point-of-care capillary glucose testing; RCT: randomized controlled trial; TIR: time in range; TBR: time below range; TAR: time above range; CV: coefficient of variation; MD: mean difference; CI: confidence interval. All outcomes are continuous and are reported as pooled mean differences (CGM − POC). Only studies providing usable outcome data were included in each pooled estimate. Certainty of evidence was assessed using the GRADE approach.

- Risk of bias: evaluated with the Cochrane RoB 2 tool; most studies were at low or some-concerns risk of bias due to open-label design and outcome measurement.
- Inconsistency: downgraded when substantial heterogeneity ($I^2 > 50\%$) could not be explained by differences in clinical context, CGM settings, or measurement definitions.
- Indirectness: downgraded for outcomes with heterogeneous definitions or incomplete reporting (mainly severe hypoglycaemia and glycaemic variability).
- Imprecision: downgraded when confidence intervals included no important effect or when event numbers were extremely low (e.g., TBR <54 mg/dL).
- Publication bias: no formal assessment performed because <10 studies contributed to each outcome; statistical tests and funnel-plot methods are unreliable under these conditions.
- Effect measure: all outcomes are continuous and summarized using inverse-variance random-effects models (REML).
- Data contribution: the number of included studies varies across outcomes because not all trials reported all endpoints.
- GRADE methodology: the assessment of the certainty of evidence was conducted in accordance with the GRADE Working Group guidelines, following the GRADE Handbook for Grading Quality of Evidence and Strength of Recommendations. Reference: SchünemannH, BrożekJ, GuyattG, et al. GRADE Handbook for Grading Quality of Evidence and Strength of Recommendations. The GRADE Working Group; 2013.

Additional notes.

- Transformations from medians/IQRs or confidence intervals to means/SDs followed the procedures outlined in the Statistical Analysis section.
- Outcomes with very low rates (especially severe hypoglycaemia) inherently showed wider confidence intervals and lower certainty ratings.
- Despite heterogeneity in some outcomes (e.g., TBR <70 mg/dL, CV%), the direction of effect consistently favored CGM for all hyperglycaemia-related metrics.

Supplementary Table S3. Glycemic thresholds for CGM-triggered alarms in the intervention arms of included studies. Thresholds for hypoglycemia and hyperglycemia alarms varied substantially across studies included in the meta-analysis. Values are shown in both mg/dL and mmol/L.

Study	Hypoglycemia Alarm	Hyperglycemia Alarm
**Olsen et al.**	<70 mg/dL (3.9 mmol/L)	>250 mg/dL (13.9 mmol/L)
**Idrees et al.**	<85 mg/dL (4.7 mmol/L)	>300 mg/dL (16.7 mmol/L)
**Spanakis et al.**	<80 mg/dL (4.4 mmol/L)	>250 mg/dL (13.9 mmol/L)
**Wang et al.**	<70 mg/dL (3.9 mmol/L)	>300 mg/dL (16.7 mmol/L)
**Thabit et al.**	<72 mg/dL (4.0 mmol/L)	>235 mg/dL (13.0 mmol/L)
**Singh et al.**	<85 mg/dL (4.7 mmol/L)	>400 mg/dL (22.2 mmol/L)
**Fortmann et al.**	<90 mg/dL (5.0 mmol/L)	>250 mg/dL (13.9 mmol/L)

RoB 2 assessment for each analyzed study

Each section is shown below together with the answer and its justification for each article analyzed.

Fortmann et al. (2020)

Domain 1: Risk of bias arising from the randomization process

- 1.1 Was the allocation sequence random? Yes. A computerized randomization system was used.
- 1.2 Was the allocation sequence concealed until participants were enrolled and assigned to interventions? Probably yes. The study describes adequate concealment.
- 1.3 Did baseline differences between intervention groups suggest a problem with the randomization process? No. The baseline characteristics were balanced.
- Risk-of-bias judgment: Low risk

Domain 2: Risk of bias due to deviations from intended interventions

- 2.1 Were participants aware of their assigned intervention during the trial? Yes. CGM is a device-based intervention, making blinding impractical.
- 2.2 Were carers and people delivering the interventions aware of participants' assigned intervention during the trial? Yes. Given the nature of CGM use, blinding of caregivers was not possible.
- 2.3 Were there deviations from the intended intervention that arose because of the trial context? No.
- 2.6 Was an appropriate analysis used to estimate the effect of assignment to intervention? Yes. The study followed an intention-to-treat (ITT) analysis.
- Risk-of-bias judgment: Some concerns

Domain 3: Risk of bias due to missing outcome data

- 3.1 Were data for this outcome available for all, or nearly all, participants randomized? Yes. The follow-up was high across both groups.
- Risk-of-bias judgment: Low risk

Domain 4: Risk of bias in measurement of the outcome

- 4.1 Was the method of measuring the outcome inappropriate? No. Glucose monitoring outcomes were objectively measured.
- 4.2 Could measurement or ascertainment of the outcome have differed between intervention groups? No.
- 4.3 Were outcome assessors aware of the intervention received by study participants? Yes.
- 4.4 Could assessment of the outcome have been influenced by knowledge of intervention received? Probably not, as glucose readings are automated and standardized.
- Risk-of-bias judgment: Low risk

Domain 5: Risk of bias in selection of the reported result

- 5.1 Were the data analyzed according to a pre-specified analysis plan? Some concerns. The study does not fully disclose all secondary outcomes.
- Risk-of-bias judgment: Some concerns

Overall Risk of Bias Judgment: Some concerns

__Singh et al. (2020)__

Domain 1: Risk of bias arising from the randomization process
- 1.1 Was the allocation sequence random? Yes. The study used a centralized system for randomization.
- 1.2 Was the allocation sequence concealed until participants were enrolled and assigned to interventions? Yes. The allocation was sealed and managed centrally.
- 1.3 Did baseline differences between intervention groups suggest a problem with the randomization process? No. Baseline characteristics were comparable.
- Risk-of-bias judgment: Low risk

Domain 2: Risk of bias due to deviations from intended interventions
- 2.1 Were participants aware of their assigned intervention during the trial? Yes.
- 2.2 Were carers and people delivering the interventions aware of participants' assigned intervention during the trial? Yes.
- 2.3 Were there deviations from the intended intervention that arose because of the trial context? No.
- 2.6 Was an appropriate analysis used to estimate the effect of assignment to intervention? Yes.
- Risk-of-bias judgment: Low risk

Domain 3: Risk of bias due to missing outcome data
- 3.1 Were data for this outcome available for all, or nearly all, participants randomized? Yes.
- Risk-of-bias judgment: Low risk

Domain 4: Risk of bias in measurement of the outcome
- 4.1 Was the method of measuring the outcome inappropriate? No.
- 4.2 Could measurement or ascertainment of the outcome have differed between intervention groups? Some concerns.
- Risk-of-bias judgment: Some concerns

Domain 5: Risk of bias in selection of the reported result
- 5.1 Were the data analyzed according to a pre-specified analysis plan? Yes.
- Risk-of-bias judgment: Low risk

Overall Risk of Bias Judgment: Low risk

__Spanakis et al. (2022)__

Domain 1: Risk of bias arising from the randomization process
- 1.1 Was the allocation sequence random? Some concerns. The study did not clearly describe the method used for randomization.
- 1.2 Was the allocation sequence concealed until participants were enrolled and assigned to interventions? Probably yes.
- 1.3 Did baseline differences between intervention groups suggest a problem with the randomization process? No.
- Risk-of-bias judgment: Some concerns

Domain 2: Risk of bias due to deviations from intended interventions
- 2.1 Were participants aware of their assigned intervention during the trial? Yes.

- 2.2 Were carers and people delivering the interventions aware of participants' assigned intervention during the trial? Yes.
- 2.3 Were there deviations from the intended intervention that arose because of the trial context? No.
- 2.6 Was an appropriate analysis used to estimate the effect of assignment to intervention? Yes.
- Risk-of-bias judgment: Low risk

Domain 3: Risk of bias due to missing outcome data
- 3.1 Were data for this outcome available for all, or nearly all, participants randomized? Some concerns due to missing CGM data in some participants.
- Risk-of-bias judgment: Some concerns

Domain 4: Risk of bias in measurement of the outcome
- 4.1 Was the method of measuring the outcome inappropriate? No.
- 4.2 Could measurement or ascertainment of the outcome have differed between intervention groups? Some concerns due to open-label design.
- Risk-of-bias judgment: Some concerns

Domain 5: Risk of bias in selection of the reported result
- 5.1 Were the data analyzed according to a pre-specified analysis plan? Yes.
- Risk-of-bias judgment: Low risk

Overall Risk of Bias Judgment: Some concerns

<u>**Idrees et al. (2024)**</u>

Domain 1: Risk of bias arising from the randomization process
- 1.1 Was the allocation sequence random? Yes. The study followed a robust randomization protocol.
- 1.2 Was the allocation sequence concealed until participants were enrolled and assigned to interventions? Yes.
- 1.3 Did baseline differences between intervention groups suggest a problem with the randomization process? No.
- Risk-of-bias judgment: Low risk

Domain 2: Risk of bias due to deviations from intended interventions
- 2.1 Were participants aware of their assigned intervention during the trial? Yes.
- 2.2 Were carers and people delivering the interventions aware of participants' assigned intervention during the trial? Yes.
- 2.3 Were there deviations from the intended intervention that arose because of the trial context? No.
- 2.6 Was an appropriate analysis used to estimate the effect of assignment to intervention? Yes.
- Risk-of-bias judgment: Low risk

Domain 3: Risk of bias due to missing outcome data
- 3.1 Were data for this outcome available for all, or nearly all, participants randomized? Yes.
- Risk-of-bias judgment: Low risk

Domain 4: Risk of bias in measurement of the outcome
- 4.1 Was the method of measuring the outcome inappropriate? No.

- 4.2 Could measurement or ascertainment of the outcome have differed between intervention groups? No.
- Risk-of-bias judgment: Low risk

Domain 5: Risk of bias in selection of the reported result

- 5.1 Were the data analyzed according to a pre-specified analysis plan? Yes.
- Risk-of-bias judgment: Low risk

Overall Risk of Bias Judgment: Low risk

<u>Wang et al. (2024)</u>

Domain 1: Risk of bias arising from the randomization process

- 1.1 Was the allocation sequence random? Some concerns. The study does not describe the exact method of randomization.
- 1.2 Was the allocation sequence concealed until participants were enrolled and assigned to interventions? Probably yes.
- 1.3 Did baseline differences between intervention groups suggest a problem with the randomization process? No.
- Risk-of-bias judgment: Some concerns

Domain 2: Risk of bias due to deviations from intended interventions

- 2.1 Were participants aware of their assigned intervention during the trial? Yes.
- 2.2 Were carers and people delivering the interventions aware of participants' assigned intervention during the trial? Yes.
- 2.3 Were there deviations from the intended intervention that arose because of the trial context? Some concerns due to varying adherence to CGM.
- 2.6 Was an appropriate analysis used to estimate the effect of assignment to intervention? Yes.
- Risk-of-bias judgment: Some concerns

Domain 3: Risk of bias due to missing outcome data

- 3.1 Were data for this outcome available for all, or nearly all, participants randomized? Yes.
- Risk-of-bias judgment: Low risk

Domain 4: Risk of bias in measurement of the outcome

- 4.1 Was the method of measuring the outcome inappropriate? No.
- 4.2 Could measurement or ascertainment of the outcome have differed between intervention groups? Some concerns due to open-label design.
- Risk-of-bias judgment: Some concerns

Domain 5: Risk of bias in selection of the reported result

- 5.1 Were the data analyzed according to a pre-specified analysis plan? Some concerns. Some secondary outcomes were not clearly pre-specified.
- Risk-of-bias judgment: Some concerns

Overall Risk of Bias Judgment: Some concerns

<u>Thabit et al. (2024)</u>

Domain 1: Risk of bias arising from the randomization process

- 1.1 Was the allocation sequence random? Some concerns. Limited details provided on the exact randomization method.
- 1.2 Was the allocation sequence concealed until participants were enrolled and assigned to interventions? Probably yes.
- 1.3 Did baseline differences between intervention groups suggest a problem with the randomization process? No.
- Risk-of-bias judgment: Some concerns

Domain 2: Risk of bias due to deviations from intended interventions

- 2.1 Were participants aware of their assigned intervention during the trial? Yes.
- 2.2 Were carers and people delivering the interventions aware of participants' assigned intervention during the trial? Yes.
- 2.3 Were there deviations from the intended intervention that arose because of the trial context? Yes. Some participants had inconsistent CGM usage.
- 2.6 Was an appropriate analysis used to estimate the effect of assignment to intervention? No. The analysis method was not fully transparent.
- Risk-of-bias judgment: High risk

Domain 3: Risk of bias due to missing outcome data

- 3.1 Were data for this outcome available for all, or nearly all, participants randomized? Some concerns. A notable number of participants had missing CGM data.
- Risk-of-bias judgment: Some concerns

Domain 4: Risk of bias in measurement of the outcome

- 4.1 Was the method of measuring the outcome inappropriate? No.
- 4.2 Could measurement or ascertainment of the outcome have differed between intervention groups? Some concerns due to open-label design.
- Risk-of-bias judgment: Some concerns

Domain 5: Risk of bias in selection of the reported result

- 5.1 Were the data analyzed according to a pre-specified analysis plan? Some concerns. Some secondary outcomes were not clearly pre-specified.
- Risk-of-bias judgment: Some concerns

Overall Risk of Bias Judgment: Some concerns

<u>**Thabit et al. (2024)**</u>

Domain 1: Risk of bias arising from the randomization process

- 1.1 Was the allocation sequence random? Some concerns. Limited details provided on the exact randomization method.
- 1.2 Was the allocation sequence concealed until participants were enrolled and assigned to interventions? Probably yes.
- 1.3 Did baseline differences between intervention groups suggest a problem with the randomization process? No.
- Risk-of-bias judgment: Some concerns

Domain 2: Risk of bias due to deviations from intended interventions

- 2.1 Were participants aware of their assigned intervention during the trial? Yes.
- 2.2 Were carers and people delivering the interventions aware of participants' assigned intervention during the trial? Yes.

- 2.3 Were there deviations from the intended intervention that arose because of the trial context? Yes. Some participants had inconsistent CGM usage.
- 2.6 Was an appropriate analysis used to estimate the effect of assignment to intervention? No. The analysis method was not fully transparent.
- Risk-of-bias judgment: High risk

Domain 3: Risk of bias due to missing outcome data

- 3.1 Were data for this outcome available for all, or nearly all, participants randomized? Some concerns. A notable number of participants had missing CGM data.
- Risk-of-bias judgment: Some concerns

Domain 4: Risk of bias in measurement of the outcome

- 4.1 Was the method of measuring the outcome inappropriate? No.
- 4.2 Could measurement or ascertainment of the outcome have differed between intervention groups? Yes. The study was not blinded, introducing potential bias.
- Risk-of-bias judgment: High risk
-

Domain 5: Risk of bias in selection of the reported result

- 5.1 Were the data analyzed according to a pre-specified analysis plan? Some concerns.
- Risk-of-bias judgment: Some concerns

Overall Risk of Bias Judgment: High risk

<u>Olsen et al. (2025)</u>

Domain 1: Risk of bias arising from the randomization process

- Was the allocation sequence random? Yes. Randomization was computer-generated in blocks, ensuring unpredictability.
- 1.2 Was the allocation sequence concealed until participants were enrolled and assigned to interventions? Probably yes. The process of allocation concealment is not explicitly detailed, but the description suggests appropriate concealment procedures.
- 1.3 Did baseline differences between intervention groups suggest a problem with the randomization process? No. The baseline characteristics between groups were comparable.
- Risk-of-bias judgment: Low risk

Domain 2: Risk of bias due to deviations from intended interventions

- 2.1 Were participants aware of their assigned intervention during the trial? Yes. Due to the nature of CGM, blinding was not feasible.
- 2.2 Were carers and people delivering the interventions aware of participants' assigned intervention during the trial? Yes. CGM use required real-time data access by caregivers.
- 2.3 Were there deviations from the intended intervention that arose because of the trial context? No. Adherence to protocol was closely supervised by a specialized diabetes team.
- 2.6 Was an appropriate analysis used to estimate the effect of assignment to intervention? Yes. The study used an intention-to-treat approach.

- Risk-of-bias judgment: Some concerns

Domain 3: Risk of bias due to missing outcome data

- 3.1 Were data for this outcome available for all, or nearly all, participants randomized? Yes. The study reported complete data for the primary outcomes, with minimal attrition.
- Risk-of-bias judgment: Low risk

Domain 4: Risk of bias in measurement of the outcome

- 4.1 Was the method of measuring the outcome inappropriate? No. Outcomes were measured using CGM data and standardized glycemic metrics.
- 4.2 Could measurement or ascertainment of the outcome have differed between intervention groups? No. Both groups were monitored with validated and objective methods.
- 4.3 Were outcome assessors aware of the intervention received by study participants? Yes.
- 4.4 Could assessment of the outcome have been influenced by knowledge of intervention received? Probably not. Outcomes were derived from automated CGM data, minimizing bias.
- Risk-of-bias judgment: Low risk

Domain 5: Risk of bias in selection of the reported result

- 5.1 Were the data analyzed according to a pre-specified analysis plan? Probably yes. The analysis was consistent with the registered protocol, although not all secondary outcomes are equally detailed.
- Risk-of-bias judgment: Some concerns

Overall Risk of Bias Judgment: Some concerns

PRISMA 2020 Checklist

Reference used:

Page MJ, McKenzie JE, Bossuyt PM, Boutron I, Hoffmann TC, Mulrow CD, et al. *The PRISMA 2020 statement: an updated guideline for reporting systematic reviews*. BMJ. 2021;372:n71. doi:10.1136/bmj.n71

**Section/Topic**	**Item**	**Checklist item**	**Reported on page/section**
**TITLE**	1	Identify the report as a systematic review/meta-analysis.	Title page
**ABSTRACT**	2	Abstract structured per PRISMA guidance.	Abstract
**INTRODUCTION**	3	Rationale for the review.	Introduction
	4	Objectives or research questions.	Introduction (end)
**METHODS**	5	Eligibility criteria.	Methods – Inclusion/Exclusion Criteria
	6	Information sources.	Methods – Search Strategy
	7	Search strategy (full strategy provided).	Methods – Search Strategy
	8	Selection process.	Methods – Search Strategy
	9	Data collection process.	Methods – Data Collection and Quality Assessment
	10a	Outcomes/data items sought.	Methods – Data Collection
	10b	Other variables and assumptions.	Methods – Data Collection
	11	Risk of bias assessment methods.	Methods – Risk of Bias
	12	Effect measures.	Methods – Statistical Analysis
	13a–f	Synthesis methods, heterogeneity, sensitivity analysis.	Methods – Statistical Analysis
	14	Reporting bias assessment.	Methods – Publication Bias

Section/Topic	Item	Checklist item	Reported on page/section
	15	Certainty assessment.	Methods – Data Collection and Quality Assessment (GRADE assessment added; Summary of Findings in Supplementary Table 2)
**RESULTS**	16a	Study selection, number screened, flow diagram.	Results + Figure 1
	16b	Cite excluded studies with reasons.	Results
	17	Characteristics of included studies.	Results + Table 1
	18	Risk of bias for each study.	Results + Supplementary material
	19	Results of individual studies.	Results (Figures 2–3)
	20a–d	Results of syntheses (meta-analysis, heterogeneity, sensitivity).	Results (Figures 2–4)
	21	Reporting biases (publication bias).	Results – Publication Bias
	22	Certainty of evidence.	Results – GRADE Summary of Findings; full table in Supplementary Table 2
**DISCUSSION**	23a	General interpretation of results in context of other evidence.	Discussion
	23b	Limitations of evidence included.	Limitations
	23c	Limitations of review processes.	Limitations
	23d	Implications for practice, policy, and research.	Discussion + Conclusions
**OTHER INFORMATION**	24a	Registration information.	Not registered
	24b	Protocol availability.	No protocol prepared
	24c	Amendments to registration/protocol.	Not applicable
	25	Sources of funding.	Funding section
	26	Competing interests.	Conflict of Interest section
	27	Availability of data/materials.	Data Availability section
