# Supplementary material for: Continuous Glucose Monitoring in Non-ICU Hospitalized Adults with Type 2 Diabetes: A Systematic Review
Source: J Clin Med. 2025 Dec 20;15(1):34. doi: 10.3390/jcm15010034 (PMC12786850; doi:10.3390/jcm15010034)
Supplement: Supplementary file 1 [file jcm-15-00034-s001.zip › jcm-4018749-supplementary-PRISMA.pdf]

PRISMA 2020 Checklist

Section and Topic	Item #	Checklist item	Location where item is reported
**TITLE**			
Title	1	Identify the report as a systematic review/meta-analysis.	Title page
**ABSTRACT**			
Abstract	2	Abstract structured per PRISMA guidance.	Abstract
**INTRODUCTION**			
Rationale	3	Rationale for the review.	Introduction
Objectives	4	Objectives or research questions.	Introduction (end)
**METHODS**			
Eligibility criteria	5	Eligibility criteria.	Methods – Inclusion/Exclusion Criteria
Information sources	6	Information sources.	Methods – Search Strategy
Search strategy	7	Search strategy (full strategy provided).	Methods – Search Strategy
Selection process	8	Selection process.	Methods – Search Strategy
Data collection process	9	Data collection process.	Methods – Data Collection and Quality Assessment
Data items	10a	Outcomes/data items sought.	Methods – Data Collection and Quality Assessment
	10b	Other variables and assumptions.	Methods – Data Collection and Quality Assessment
Study risk of bias assessment	11	Risk of bias assessment methods.	Methods – Risk of Bias
Effect measures	12	Effect measures.	Methods – Statistical Analysis
Synthesis methods	13a	Synthesis methods, heterogeneity, sensitivity analysis.	Methods – Statistical Analysis
	13b	Synthesis methods, heterogeneity, sensitivity analysis.	Methods – Statistical Analysis
	13c	Synthesis methods, heterogeneity, sensitivity analysis.	Methods – Statistical Analysis
	13d	Synthesis methods, heterogeneity, sensitivity analysis.	Methods – Statistical Analysis
	13e	Synthesis methods, heterogeneity, sensitivity analysis.	Methods – Statistical Analysis
	13f	Synthesis methods, heterogeneity, sensitivity analysis.	Methods – Statistical Analysis
Reporting bias assessment	14	Reporting bias assessment.	Methods – Publication Bias
Certainty assessment	15	Reporting bias assessment.	Methods – Data Collection and Quality Assessment (GRADE assessment added; Summary of Findings in

PRISMA 2020 Checklist

Section and Topic	Item #	Checklist item	Location where item is reported
			Supplementary Table 2)
**RESULTS**			
Study selection	16a	Study selection, number screened, flow diagram.	Results + Figure 1
	16b	Cite excluded studies with reasons.	Results
Study characteristics	17	Characteristics of included studies.	Results + Table 1
Risk of bias in studies	18	Risk of bias for each study.	Results + Supplementary material
Results of individual studies	19	Results of individual studies.	Results (Figures 2–3)
Results of syntheses	20a	Results of syntheses (meta-analysis, heterogeneity, sensitivity).	Results (Figures 2–4)
	20b	Results of syntheses (meta-analysis, heterogeneity, sensitivity).	Results (Figures 2–4)
	20c	Results of syntheses (meta-analysis, heterogeneity, sensitivity).	Results (Figures 2–4)
	20d	Results of syntheses (meta-analysis, heterogeneity, sensitivity).	Results (Figures 2–4)
Reporting biases	21	Reporting biases (publication bias).	Results – Publication Bias
Certainty of evidence	22	Certainty of evidence.	Summary of Findings; full table in Supplementary Table 2
**DISCUSSION**			
Discussion	23a	General interpretation of results in context of other evidence.	Discussion
	23b	Limitations of evidence included.	Limitations
	23c	Limitations of review processes.	Limitations
	23d	Implications for practice, policy, and research.	Discussion + Conclusions
**OTHER INFORMATION**			
Registration and protocol	24a	Registration information.	Not registered
	24b	Protocol availability.	No protocol prepared
	24c	Amendments to registration/protocol.	Not applicable
Support	25	Sources of funding.	Funding section
Competing interests	26	Competing interests.	Conflict of Interest section
Availability of data, code and other materials	27	Availability of data/materials.	Data Availability section

From: Page MJ, McKenzie JE, Bossuyt PM, Boutron I, Hoffmann TC, Mulrow CD, et al. The PRISMA 2020 statement: an updated guideline for reporting systematic reviews. BMJ 2021;372:n71. doi: 10.1136/bmj.n71. This work is licensed under CC BY 4.0. To view a copy of this license, visit https://creativecommons.org/licenses/by/4.0/
